# Efficacy of Clinical Tests in the Diagnosis of Meralgia Paresthetica: A Case Control Study

**DOI:** 10.1155/carm/5191280

**Published:** 2024-12-09

**Authors:** Shasthara Paneyala, Nemichandra S. Chandrashekhar, Harsha Sundaramurthy, Akila Prashant, Lakshmi Krishna

**Affiliations:** ^1^Department of Neurology, JSS Medical College, JSS Academy of Higher Education and Research, Mysuru, India; ^2^Department of Biochemistry, JSS Medical College, JSS Academy of Higher Education and Research, Mysuru, India; ^3^Department of Medical Genetics, JSS Medical College, JSS Academy of Higher Education and Research, Mysuru, India

**Keywords:** meralgia, neurodynamic testing, pelvic compression test, Tinel's sign

## Abstract

**Background:** Meralgia paresthetica (MP), a common entrapment syndrome, presents with paresthesias in the anterolateral aspect of the thigh. Clinical tests used to diagnose MP are the pelvic compression test, neurodynamic testing, and Tinel's sign. The diagnostic accuracy of these three tests has not been analyzed to date. Hence, this study aims to analyze the sensitivity and specificity of these clinical tests.

**Case Study:** This study was a hospital-based case-control study that included 30 electrophysiologically proven patients with MP. The data were gathered over a period of 6 months after obtaining institutional ethics committee clearance. Demographics and risk factors among our cases are in keeping with the global scenario. The sensitivity and specificity of the pelvic compression test were 86.7% and 93.0%, the Tinsel sign was 85.1% and 87.5%, and neurodynamic testing was 86.7% and 93.0%, respectively.

**Conclusion:** Our study proves that these tests are a simple and accurate aid in the diagnosis of MP. These bedside clinical tests can be utilized to establish a diagnosis of MP in a setting of conflicting differentials such as lumbar canal stenosis or thoracolumbar junction disc pathologies and guide in choosing the next line of investigation.

## 1. Introduction

Meralgia paresthetica (MP), a common lower limb entrapment syndrome, has a reported incidence rate of 4.3 cases per 10,000 patient years [1]. It presents with pain, paresthesias, and occasionally sensory loss in the anterolateral aspect of the thigh, in the distribution of the lateral cutaneous nerve of the thigh (LCFN) [2]. It was first described in 1885 by a neuropathologist Martin Bernhardt, and the term MP was coined by Vladimir Karlovich Roth, hence also referred to as the Bernhardt_Roth syndrome [3]. The LFCN is a sensory branch of the lumbar plexus and has a root value of L1 to L3. It enters the thigh through a tunnel formed by the inguinal ligament and the anterior superior iliac spine which is the most common site of entrapment [4, 5]. Factors implicated in the causation of MP include obesity (BMI ≥ 30), pregnancy, direct trauma, muscle spasm, scoliosis, iliacus hematoma, leg length changes, diabetes mellitus (DM), alcoholism, and lead poisoning [6–11]. Clinical presentations are highly diverse and include varying combinations of burning, numbness, and lightning pains; however, the predominant symptom will be constant in the patient [11]. Clinical tests used to diagnose MP include pelvic compression test, neurodynamic testing, and Tinel's sign [12]. Among these clinical tests, only the sensitivity and specificity of the pelvic compression test were assessed in a study of 20 patients [13]. The diagnostic accuracy of the other two tests in MP has not been analyzed to date. The gold standard to confirm the diagnosis of MP is electrophysiological testing, i.e., the somatosensory-evoked potentials and the sensory nerve conductions [14–16].

The challenge in making the diagnosis lies in the fact that certain diseases such as lumbar stenosis, lumbar radiculopathies, and disc compression can have clinical presentations similar to MP [17]. Despite significant incidence rates and presence of close clinical diagnostic differentials, there is a paucity of studies aimed at specifically exploring the diagnostic efficacy of clinical techniques. Hence, this study aims to analyze the utility of clinical tests in the diagnosis of MP. In addition, we also aim to analyze the demographics, risk factors, and clinical features in comparison to the global scenario.

### 1.1. Patient Consent Statement

Written informed consent was obtained from all participants prior to their inclusion in the study, in compliance with ethical guidelines.

## 2. Case Presentation

This study was a hospital-based case-control study that included 30 electrophysiologically proven patients with MP who reported to our outpatient department. Controls were age- and sex-matched patients who had sensory symptoms or pain in the anterior/lateral or anterolateral thigh secondary to alternate causes and normal LFCN conduction studies. The data were gathered over six months from July to December 2020 after obtaining institutional ethics committee clearance.

After obtaining written informed consent from the study participants, descriptive analysis of symptomology, underlying risk factors, and demographic details were collected and entered into a prestructured proforma. Detailed neurological examination was done, and three clinical tests were performed.

The pelvic compression test is performed by positioning the patient on his side with the symptomatic side facing up and application of a downward, compression force on the pelvis for 45 s. The test is positive if the patient experiences a reduction in symptoms [13]. The neurodynamic test is also carried out with the patients on his side and the symptomatic side up. The bottom knee is bent and after stabilizing the pelvis, the hip is adducted. The test is considered to be positive if this maneuver reproduces the patient's symptoms [12].

Tinel's sign is elicited by tapping just below the anterior superior iliac spine and considered to be positive if paresthesias occur [12]. SPSS version 24.0 for Windows was used for the testing of data. The confidence interval and significance level for this study considered was 95% and *p* < 0.05, respectively. Percentage and ratio were included in the descriptive statistics of the data. A chi-square test was applied to analyze the sensitivity and specificity of each clinical test.

In our study, 30 patients with electrodiagnostically proven cases of meralgia were selected along with age-matched controls. The mean age among the cases was 59.57 (±6.15) years with the majority in the age group of 51–60 years (56.7%). The youngest patient was 48 years old, while the oldest patient was 74 years old. The mean age among the controls was 59.7 (±6.15). Among both the cases and controls, the ratio of men to women was 1:1, i.e., 15 patents each (Tables 1 and 2).

Among the cases, 12 (30%) patients had BMI between 23.0–24.9 and 3 (10%) had BMI ≥ 25 falling into the overweight and obese category, respectively. There was no patient with a history of recent weight fluctuations. The patients with DM among the cases and controls were 12 (40%) and 9 (30%), respectively. Hypothyroidism was present among 6 (20%) of the cases and 4 (13.33%) of the controls, 27 of the cases (90%) had unilateral symptoms while the rest 3 (10%) had bilateral symptoms. The majority of the patients (20/66.66%) had sensory paresthesias in the lateral aspect of the thigh, while a few (9/30%) had symptoms on the anterolateral aspect and one patient (3.3%) had symptoms only on the anterior aspect. The prominent symptom presentation was a sensation of burning seen in 15 (50%) cases, followed by coldness, deep muscle pain, numbness, and lightening pains in 5 (16.7%), 4 (13.3%), 3 (10%), and 3 (10%), respectively. Twelve patients (40%) had relief in symptoms on sitting, while the rest did not report any change in symptom characteristics.

Among the 30 controls, the various etiologies for symptom causation included lumbar canal stenosis, L3 and L4 radiculopathy, osteoarthritis of the hip and knee, pyriformis syndrome, and ischial bursitis in 10 (33.3%), 9(30), 6(20%), 3(10%), and 2(6.6%), respectively.

Among the cases, the mean amplitude, side to side variability, and conduction velocity were 1.34 ± 0.81 *μ*V, 34.73 ± 1.76, and 34.57 ± 10.54 m/s, respectively. All the controls had normal values, and the corresponding parameters among the controls were 4.46 ± 0.77 *μ*V, 21.4 ± 5.09, and 52.03 ± 2.06 m/s, respectively (Table 3).

The pelvic compression test was positive in 28 (86.7%) of the cases and 2 (6.6%) of the controls. Tinel's sign was positive in 24 (80%) of the cases and 4 (13.3%) of the controls. Neurodynamic testing was positive in 28 (86.7%) of the cases and 2 (6.6%) of the controls (Table 4).

The pelvic compression test was found to have a sensitivity of 86.7% and specificity of 93.3%. The test has a positive predictive value of 92.9% and negative predictive value of 87.5%. The test and the gold standard, i.e., nerve conduction studies agree on 54 out of 60 having a diagnostic accuracy of 90%. The kappa value of 0.8 indicates very good agreement with a *p* value of < 0.001.

Tinel's sign was found to have a sensitivity of 85.71% and specificity of 87.50%. The test has a positive predictive value of 85.71% and negative predictive value of 87.50%. The test and the gold standard agree on 52 out of 60 having a diagnostic accuracy of 90%. The kappa value of 0.8 indicates very good agreement with a *p* value of < 0.001.

Neurodynamic testing was found to have a sensitivity of 86.7% and specificity of 93.3%. The test has a positive predictive value of 92.9% and a negative predictive value of 87.5%. The test and the gold standard agree on 54 out of 60, having a diagnostic accuracy of 86.67%. The kappa value of 0.8 indicates very good agreement with a *p* value of < 0.001 (Table 5).

## 3. Discussion

Our study was aimed to assess the accuracy of specific clinical tests in the diagnosis of MP. In our study, the majority of the cases were noted to be in 51–60 years of age. This is consistent with the global profile of MP with most cases occurring in the range of 30–65 years [1, 18]. In our study, there were an equal number of males and females. Most studies find a male predominance [19, 20]; however, a case-control study conducted to specifically assess the demographics of the disease noted a female predominance [1].

Most studies indicate a clear association between obesity and MP [6, 19, 21, 22]. However, in one case-control study conducted in 2004, obesity was not identified as a significant risk factor. As per the revised BMI cutoff for Indians [23], a total of 12 patients had an increased BMI with 30% and 10% falling into the overweight and obese categories, respectively. However, we did not find a significant association between BMI and MP. The relation between weight and causation of MP is a complex one, as not only obesity but fluctuations in weight have also been proven to be a risk factor [19]. None of our patients had a history of significant weight fluctuations.

The prevalence of DM among our cases and controls was 40% and 30%, respectively. In a population-based study conducted in 2011, it was found that 28% of the patients with MP and 17% of the controls had DM. It also showed that patients with MP were twice as likely to develop DM [22]. In our study, despite there being a higher percentage of diabetes and hypothyroidism among the cases, there was no statistically significant difference noted.

Major risk factors implicated in the causation of MP are DM, hypothyroidism, and obesity [1, 12]. These factors increase the susceptibility of the nerve to entrapment under the inguinal ligament. Other risk factors commonly implicated in the causation of MP, such as recent pregnancy, use of steroids, and specific garments predisposing to a compression such as a military uniform, trauma, or lower abdominal surgeries, were not encountered in our study. Among the 30 patients, 12 patients (40%) did not have any discernible risk factors. This is substantially lower than a population-based study, which found that 79% of their patients did not have an identifiable risk factor for MP [1].

Our patients presented with symptom duration varying from 1 month to 2 years. A study on MP conducted in 1991 showed a mean duration of 2 months–6 years [24]. However, a recent study conducted on 120 patients with meralgia found a wider duration of 2 weeks–20 years. In this study, only 47 of the patients were referred with a clinical diagnosis of MP, while the majority had been misdiagnosed to have a variety of other diseases [25]. Among our patients, 27, i.e., 90% had unilateral symptoms. This is consistent with most studies where 10%–20% of the cases were bilateral [2, 19, 22].

All 30 patients had sensory symptoms of varying severity in the typical distribution of the LCFN, i.e., 66.66% in the lateral and 30% in the anterolateral aspect of the thigh, except for one patient who had symptoms localized to the anterior aspect of the thigh only. This is comparable to a study carried out on MP, in which 73.3% of the patients had symptoms in the lateral aspect and 26.7% had symptoms in the anterolateral part of the thigh [25]. The anterior symptom presentation is attributed to the anastomosis between the superior perforator nerve, inferior perforator nerve, and the LFCN [16]. Hence, it is always prudent to consider the possibility of MP despite exclusive anterior thigh sensory symptoms.

The major sensory symptoms among our patients in descending order of occurrence were a sensation of burning followed by coldness, dull pain, numbness, and lightening pains consistent with the current literature [12, 25]. A small number of patients (16.7%) complained of a sensation of coldness in the area. This symptomology is explained by the presence of efferent sympathetic fibers within the LFCN [26].

In addition, a large proportion of patients (40%) reported attenuation of symptoms on prolonged sitting. This temporary relief occurs due to the relaxation of the inguinal ligament and has been documented in the literature [12, 27]. While this finding is a common feature of MP, it can also be seen in other conditions mimicking MP and hence is of no diagnostic significance when used in isolation. However, in combination with typical location and other supportive clinical features, this finding can be of diagnostic relevance.

All 30 of our patients had classic electrophysiological findings, with a mean amplitude, side to side variability, and conduction velocity of 1.34 ± 0.81 microvolts, 34.73 ± 1.76, and 34.57 ± 10.54 m/s, respectively, fitting the diagnostic criteria [14, 25, 28, 29]. Even among the patients with bilateral disease, there was clear clinical and electrophysiological asymmetry in keeping with findings from previous studies [12, 25].

None of our patients had elicitable motor and sensory or reflex abnormalities. The pelvic compression test, Tinel's sign, and neurodynamic testing were positive in 28 (86.7%), 24 (80%), and 28 (86.7%) of the cases, respectively. Pelvic compression test had a sensitivity and specificity of 86.7% and 93.3%, respectively, in our study. In a study conducted on 45 patients with MP, a higher sensitivity (95%) but a similar specificity (93.3%) was computed, which was a unique study in MP specifically assessing the diagnostic efficacy of a clinical test [13].

In our study, Tinel's sign had a sensitivity and specificity of 85.1% and 87.50%, respectively. This test was performed by tapping adjacent to the anterior superior iliac spine and eliciting characteristic paresthesias. Neurodynamic testing had a sensitivity and specificity of 86.7% and 93.3%, respectively. These two tests have been frequently used in the diagnosis of MP [12, 30–32]; however, no study has analyzed the sensitivity and specificity of this test. The sensitivity and specificity of all the three tests were statistically significant.

Among the controls, 19 patients (63%) had spinal cord and radicular pathologies, while the rest had musculoskeletal diseases, causing isolated anterolateral sensory symptoms. It is well known that cord pathologies involving the thoracolumbar junction have atypical presentations due to transition from UMN to LMN i.e., epiconus, conus to cauda equina, thus leading to frequent delay in the diagnosis [33, 34]. In a retrospective study of 26 patients with thoracolumbar junction disc herniations, it was noted that the ones with a pathology at L1 to L2 had presentations very similar to MP with no motor or reflex changes to guide the diagnostic process [35].

Against this backdrop, it is of paramount importance to formulate a clinical algorithm in order to guide the next step in the diagnostic workup of such cases. When the three tests are positive, it will act as an indicator of underlying MP, guiding the clinician to initiate electrophysiological testing. This is specifically valid in the context of atypical findings such as symptoms restricted to the anterior aspect of the thigh, vague sudomotor symptoms, or nonradiating back pain. Conversely, if all these three tests are negative, possibility of a thoracolumbar junction disc pathology or based on the clinical setting, a musculoskeletal disease can be thought of. In the latter context, the next line of investigation would be an MRI. This systematic algorithm will streamline the diagnostic process and save precious resources, both in terms of time and money. The therapeutic implications are twofold. Firstly, the management of MP varies greatly from its differentials. Most of the patients with MP respond well to weight loss, control of metabolic parameters, and neuropathic pain medications [25, 36, 37]. Secondly, if surgery is performed on a patient with a coexistent spinal pathology and MP is not dealt with, the patient will have persistent symptoms post procedure. Hence, when confronted with a possible thoracolumbar junction disc pathology, performing these tests would add to the diagnostic clarity.

All 30 of our patients are on regular follow-up and have responded well to the medical line of treatment alone. Since there is a paucity of data on the need for surgical intervention in MP [36] and our patients have had symptom reduction with a conservative approach, the same has been continued. We acknowledge that formal sample size calculations were not performed for this study, and we have noted this as a limitation, emphasizing the need for larger multicentric studies to confirm the diagnostic efficacy of the clinical tests.

This study was designed to quantify the diagnostic yield from three clinical tests in an attempt to form a predictive clinical algorithm for detecting MP. To our knowledge, it is the only such study assessing the sensitivity and specificity of three clinical tests in the diagnosis of MP. Application of these tests, especially when faced with a diagnostic uncertainty, can guide the decision process to choose the next line of investigation. Our study proves that these tests are a simple, handy, and accurate aid in the diagnosis of MP with far-reaching therapeutic implications. In an age where investigative tools have streamlined diagnostics, the relevance of a clinical diagnosis must not be forgotten [38].

## Figures and Tables

**Table 1:** Age distribution among cases and controls.

Age	Group		Total *n* (%)	Statistical analysis
	Control *n* (%)	Case *n* (%)		
< 50	2 (6.7)	2 (6.7)	4 (6.7)	Pearson chi-square value = 1.504 Df = 3 *p* value = 0.681
51–60	17 (56.7)	16 (53.3)	33 (55.0)	
61–70	8 (26.7)	11 (36.7)	19 (31.7)	
> 70	3 (10.0)	1 (3.3)	4 (6.7)	

Total	30 (100.0)	30 (100)	60 (100)	

**Table 2:** Sex distribution among cases and controls.

Sex	Group		Total *n* (%)	Statistical analysis
	Control *n* (%)	Case *n* (%)		
Female	15 (50.0)	15 (50.0)	30 (50.0)	Pearson chi-square value = 0.000 Df = 1 *p* value = 1.000
Male	15 (50.0)	15 (50.0)	30 (50.0)	

Total	30 (100.0)	30 (100)	60 (100)	

**Table 3:** Electrophysiological findings of LFCN among cases and controls.

Parameter	Cases (*n* = 30)	Controls (*n* = 30)	*T*	*p* value
	Mean ± sd	Mean ± sd		
Amplitude (*μ*V)	1.34 ± 0.81	4.46 ± 0.77	−15.328	< 0.001
Side to side variability	34.73 ± 1.76	21.4 ± 5.09	13.561	< 0.001
Conduction velocity (m/s)	34.57 ± 10.54	52.03 ± 2.06	−8.906	< 0.001

**Table 4:** Clinical tests among cases and controls.

Parameter	True negative	True positive	False negative	False positive
Pelvic compression test	28	26	4	2
Neurodynamic testing	28	26	4	2
Tinel sign	28	26	4	2

**Table 5:** Diagnostic accuracy of clinical tests.

Parameter	Sensitivity (%)	Specificity (%)	Positive predictive value (%)	Negative predictive value (%)	Diagnostic accuracy (%)	*p* value
Pelvic compression test	86.70	93.30	92.90	87.50	90.00	< 0.001
Tinel sign	85.71	87.50	92.90	87.50	86.67	< 0.001
Neurodynamic testing	86.70	93.30	92.90	87.50	90.00	< 0.001

## Data Availability

The datasets analyzed during the current study are available from the corresponding author upon reasonable request. In addition, comprehensive literature sources used for the literature review are cited appropriately within the manuscript.
